# Development and validation of a simple and practical model for early detection of diabetic macular edema in patients with type 2 diabetes mellitus using easily accessible systemic variables

**DOI:** 10.1186/s12967-024-05328-y

**Published:** 2024-05-31

**Authors:** Guanrong Wu, Yijun Hu, Qibo Zhu, Anyi Liang, Zijing Du, Chunwen Zheng, Yanhua Liang, Yuxiang Zheng, Yunyan Hu, Lingcong Kong, Yingying Liang, Maman Lawali Dan Jouma Amadou, Ying Fang, Yuejuan Liu, Songfu Feng, Ling Yuan, Dan Cao, Jinxin Lin, Honghua Yu

**Affiliations:** 1 Department of Ophthalmology, Guangdong Provincial People’s Hospital (Guangdong Academy of Medical Sciences), Guangdong Eye Institute, Southern Medical University, Guangzhou, China; 2 Department of Endocrinology, Guangdong Provincial People’s Hospital, Guangdong Academy of Medical Sciences, Guangzhou, China; 3 School of Medicine, South China University of Technology, Guangzhou, China; 4 Department of Ophthalmology, The People’s Hospital of JiangMen, Jiangmen, China; 5 Department of Ophthalmology, The First Affiliated Hospital of Kunming Medical University, Kunming, China; 6 Department of Ophthalmology, Zhujiang Hospital of Southern Medical University, Guangzhou, China; 7 Guangdong Provincial Key Laboratory of Artificial Intelligence in Medical Image Analysis and Application, Guangzhou, China

**Keywords:** Diabetic macular edema, Prediction model, Risk score, Risk factor, Type 2 diabetes mellitus

## Abstract

**Objective:**

Diabetic macular edema (DME) is the leading cause of visual impairment in patients with diabetes mellitus (DM). The goal of early detection has not yet achieved due to a lack of fast and convenient methods. Therefore, we aim to develop and validate a prediction model to identify DME in patients with type 2 diabetes mellitus (T2DM) using easily accessible systemic variables, which can be applied to an ophthalmologist-independent scenario.

**Methods:**

In this four-center, observational study, a total of 1994 T2DM patients who underwent routine diabetic retinopathy screening were enrolled, and their information on ophthalmic and systemic conditions was collected. Forward stepwise multivariable logistic regression was performed to identify risk factors of DME. Machine learning and MLR (multivariable logistic regression) were both used to establish prediction models. The prediction models were trained with 1300 patients and prospectively validated with 104 patients from Guangdong Provincial People’s Hospital (GDPH). A total of 175 patients from Zhujiang Hospital (ZJH), 115 patients from the First Affiliated Hospital of Kunming Medical University (FAHKMU), and 100 patients from People’s Hospital of JiangMen (PHJM) were used as external validation sets. Area under the receiver operating characteristic curve (AUC), accuracy (ACC), sensitivity, and specificity were used to evaluate the performance in DME prediction.

**Results:**

The risk of DME was significantly associated with duration of DM, diastolic blood pressure, hematocrit, glycosylated hemoglobin, and urine albumin-to-creatinine ratio stage. The MLR model using these five risk factors was selected as the final prediction model due to its better performance than the machine learning models using all variables. The AUC, ACC, sensitivity, and specificity were 0.80, 0.69, 0.80, and 0.67 in the internal validation, and 0.82, 0.54, 1.00, and 0.48 in prospective validation, respectively. In external validation, the AUC, ACC, sensitivity and specificity were 0.84, 0.68, 0.90 and 0.60 in ZJH, 0.89, 0.77, 1.00 and 0.72 in FAHKMU, and 0.80, 0.67, 0.75, and 0.65 in PHJM, respectively.

**Conclusion:**

The MLR model is a simple, rapid, and reliable tool for early detection of DME in individuals with T2DM without the needs of specialized ophthalmologic examinations.

**Supplementary Information:**

The online version contains supplementary material available at 10.1186/s12967-024-05328-y.

## Introduction

Diabetic macular edema (DME) is the primary cause of vision loss in patients with type 2 diabetes mellitus (T2DM) [1]. The prevalence of DME in T2DM patients ranges from 1.4–12.8% [2], which is estimated to affect more than 20 million patients worldwide [1]. With the rapid rise in the number of individuals with diabetes, the disease burden of DME has increased exponentially [3, 4]. Moreover, DME patients who fail to get timely treatments may suffer from irreversible visual impairment that severely impacts the quality of life and imposes a significant financial burden [5, 6]. Thus, early diagnosis of DME is crucial for rational risk stratification, early strategy management, curative effect optimization, as well as reduction of health care costs [7–9].

Early and regular fundus examinations are recommended for DM patients, but it arises challenges in terms of medical-ophthalmic referrals and patient adherence, and often unavailable in local and remote areas [10, 11]. There is also a growing shortage of ophthalmologists in many countries, especially the developing countries [10, 12–14]. Meanwhile, because DM is largely managed by physician internists, cross-department diagnosis and treatment pose a major challenge for DME management [15]. Therefore, there is an urgent need for a simple, rapid, reliable, and cost-effective DME assessment tool in the community-based clinical practice which does not depend on ophthalmic specialists or devices but on easily accessible systemic variables. The application of this prediction model would not only increase the efficiency and accessibility of DME screening but also improve the prognosis and saves medical resources.

The assessment of systemic risk factors for DME is beneficial for monitoring disease status, however, these factors remain unclear and inconsistent among various studies. Duration of DM, glycemic control, hypertension, dyslipidemia, obesity, nephropathy, anemia, sleep apnea, glitazone usage, pregnancy, as well as genetic predisposition, were reported to be associated with DME [1, 16, 17]. Moreover, although some previous studies have proposed risk evaluation models for DME [18–20], most of them were limited by their small sample sizes, lack of diabetes type classification, use of uneasily-accessible complicated systemic variables, and lack of external validation, which hinder their application in clinical practice, especially in community hospital or remote areas where ophthalmic investigations are not readily available.

Therefore, in the present study, we aim to develop a simple and convenient DME risk prediction tool using systemic variables easily obtained by non-ophthalmic specialists, in order to rapidly screen DME in T2DM patients.

## Methods

### Design and population of the study

Patients diagnosed with T2DM in the Endocrinology Department and underwent ophthalmic consultation in the Ophthalmology Department of Guangdong Provincial People’s Hospital (GDPH) from January 2017 to August 2022, the Zhujiang Hospital of Southern Medical University (ZJH) from January 2016 to January 2022, the First Affiliated Hospital of Kunming Medical University (FAHKMU) from January 2018 to January 2022, and the People’s Hospital of JiangMen (PHJM) from January 2019 to January 2022 were recruited in this study.

Patients were included if they were diagnosed with T2DM according to the 2015 American Diabetes Association (ADA) criteria [21] and had a DR and DME screening. DME was defined as having any retinal thickening or hard exudates within one disc diameter from the center of the fovea in the presence of DR features, according to the ETDRS report [22]. The exclusion criteria for patients were (1) under 18 years old; (2) inadequate quality of fundus photographs; (3) missing data on systemic variables; (4) complicated with serious systemic diseases, including immunodeficiency diseases and malignant diseases; (5) macular edema secondary to causes other than DR, such as age-related macular degeneration, polypoidal choroidal vasculopathy, retinal artery/vein occlusion, and rhegmatogenous retinal detachment; (6) with history of DR treatments within 6 months, including anti-vascular endothelial growth factor (VEGF) injection, retinal laser therapy, or intraocular surgery; (7) pregnancy.

### Ophthalmic examinations

All patients underwent comprehensive ophthalmic examinations, including best-corrected visual acuity (BCVA) (measured on a decimal chart and presented as logMAR), autorefraction, intraocular pressure, slit-lamp examination, and fundus photography (non-stereoscopic 45° photograph of the central fundus and of the optic disc) using a non-mydriatic retinal camera (Topcon TRC; Topcon, Tokyo, Japan). Based on medical records and fundus images, we performed DR and DME assessments by two ophthalmologists (Wu GR, Du ZJ), and any inconsistency would be judged by a retinal specialist (Hu YY).

### Systemic data collection

Medical history and laboratory data during hospitalization were collected. The basic information included age, sex, BMI, systolic blood pressure (SBP), diastolic blood pressure (DBP), history of hypertension, anti-hypertensive drug use, history of cardiovascular disease, duration of DM, and treatment of DM. The BMI was calculated as weight in kilograms divided by the square of height in meters. Laboratory tests included blood glucose test [HbAlC levels], blood lipid test [triglycerides (TG), total cholesterol (TC), low-density lipoprotein cholesterol (LDLc), high-density lipoprotein cholesterol (HDLc)], blood routine examination [red blood cells (RBC), hemoglobin (Hb), hematocrit (HCT), white blood cells (WBC), platelet count (PLT)], renal function tests [blood urea nitrogen (BUN), uric acid (UA), serum creatinine (Scr), urinary albumin, and urinary creatinine (Ucr), urine albumin-to-creatinine ratio (UACR)]. UACR was divided into 3 stages based on the definition of the USA National Kidney Foundation [23]: Stage 1 of UACR (normal or low albuminuria, UACR < 30 mg/g), Stage 2 of UACR (microalbuminuria, UACR: 30-300 mg/g), Stage 3 of UACR (macroalbuminuria, UACR ≥ 300 mg/g). The value of the estimated glomerular filtration rate (eGFR) was calculated using the CKD-EPI creatinine equation as follows [24]: *141×min (Scr/κ,1)^α × max (Scr/κ,1)^-1.209 × 0.993^Age×1.018(if female)×1.159(if black)*, in which Scr is serum creatinine in mg/dL, κ is 0.7 for females and 0.9 for males, α is -0.329 for females and − 0.411 for males, min indicates the minimum of Scr/κ or 1, and max indicates the maximum of Scr/κ or 1.

### Development and validation of DME prediction model

Patients from GDPH from January 2017 to March 2022 were assigned to a training set, and patients from May 2022 to August 2022 were used as prospective validation set. The data sets of ZJH, FAHKMU, and PHJM were used for external validation. Baseline characteristics of datasets were expressed as frequency (percentage) for categorical variables and means ± SDs for continuous variables. We compared the characteristics of patients between the DME group and non-DME group in the training set using the Chi-square tests for categorical variables and the t-tests for continuous variables. Univariate logistic regressions were applied to identify risk factors for DME, and variables significantly associated with DME (*P* < 0.250) were included in the forward stepwise multivariable logistic regression (FSMLR) model. Variables with P values < 0.05 in the MLR model were considered candidate variables for DME prediction. In order to ensure the rationality of variables, we selected systematic variables based not only on multivariable logistic regression but also on the expert opinions of ophthalmologists and endocrinologists, considering their clinical significance, accessibility, and previous research findings [17, 25, 26]. In addition, we constructed 5 machine learning models support Vector Mac (SVM); random forest (RF); extreme gradient boosting (XGB); multilayer perceptron (MLP); adaptive Boosting (ADABOOST); using all variables to identify DME. Five-fold cross-validation was applied for internal validation in the training set. Finally, we selected the best performing model in internal validation as the final DME prediction model for prospective validation from GDPH and external validation from ZJH, FAHKMU, and PHJM.

### DME risk score

The selected systematic variables were adopted in the construction of the risk score for DME in T2DM patients using the Framingham score function. The risk score units were derived by approximating the regression coefficient values to the nearest whole number as a weighted scale. The overall risk score was calculated by summation of the individual risk score units. Each total score could be corresponded to the estimated risk of DME. The predictive power of the DME risk score and its association with DME were evaluated in the validation set.

### Statistical analysis

To statistically analyze the performance of the prediction models and risk score in predicting DME status, we evaluated the area under the receiver operating characteristic curve (AUC), accuracy (ACC), sensitivity, and specificity. These processes were performed in all the datasets. All statistical analyses were conducted using Stata version 16.0 (StataCorp, College Station, TX) and Python (version 3.7.0, Python Software Foundation). Double-sided P-value < 0.05 was considered to be statistically significant.

## Results

### Participant characteristics

**Fig. 1:**
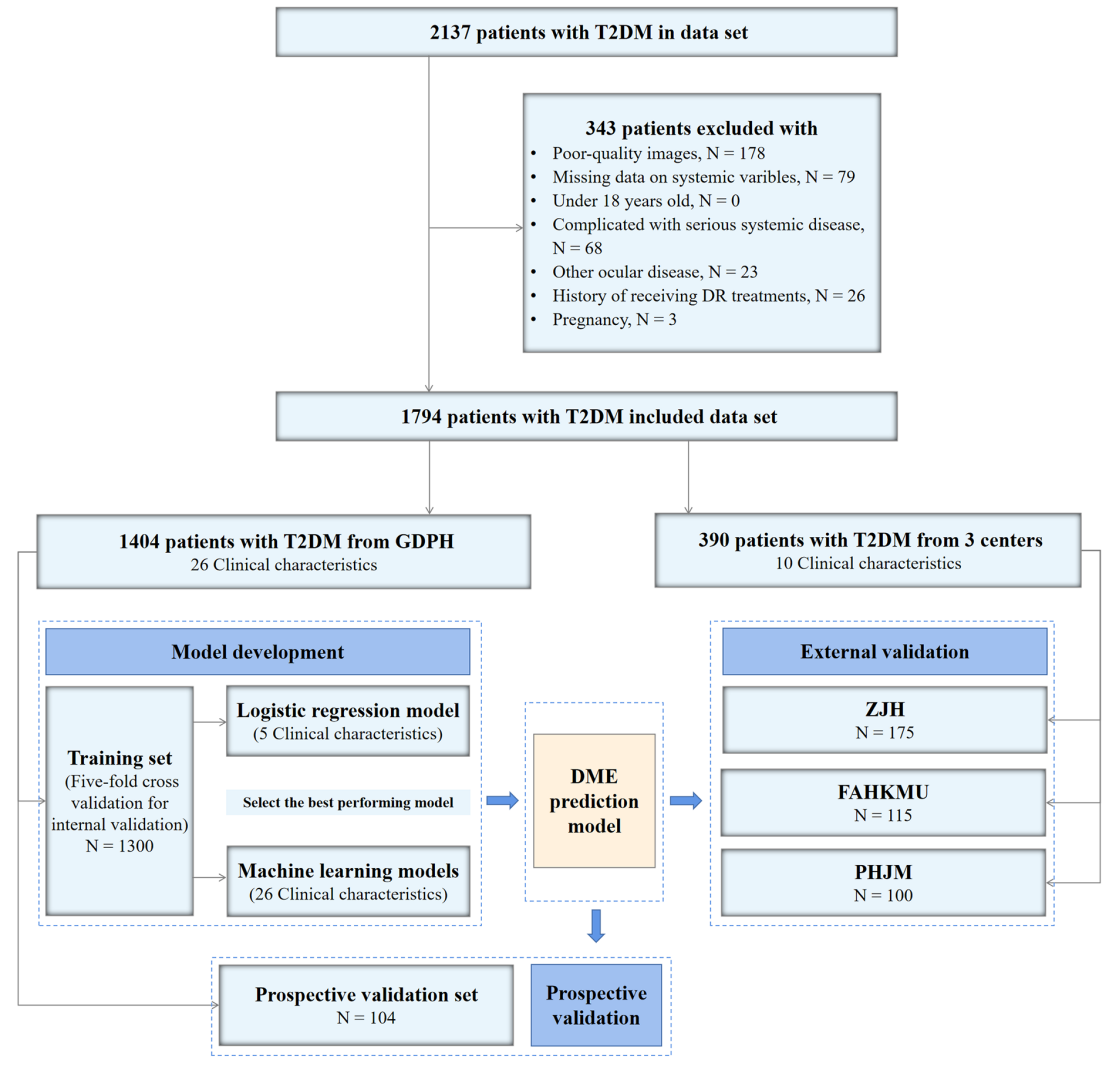
The Flowchart of developing a DME prediction model for T2DM patients based on systemic variables. Abbreviations: DME, diabetic macular edema; T2DM, Type 2 diabetes mellitus; DR, diabetic retinopathy; GDPH, the Guangdong Provincial People’s Hospital; ZJH, the Zhujiang Hospital of Southern Medical University; PHJM, People’s Hospital of JiangMen; FAHKMU, the First Affiliated Hospital of Kunming Medical University

As shown in Fig. 1, 2137 patients with T2DM were initially collected from the four centers. After excluding patients with poor-quality images (*n* = 178), missing data on systemic variables (*n* = 79), serious systemic diseases (*n* = 68) and other ocular diseases (*n* = 23), history of receiving DR treatments within 6 months (*n* = 26), and pregnancy (*n* = 3), 1794 patients with T2DM were eligible and included in this study. We used the training set (1300 patients from GDPH) to develop the DME prediction models and the DME risk score, and used other datasets for validation. Table 1 presented the baseline characteristics of each dataset. The associations between visual acuity and DR grade with DME, and between systemic variables and DR are shown in Supplementary Tables S1 and S2.

**Table 1:** Baseline characteristics of data sets from four centers

	Training set	Prospective validation	External validation		
	GDPH	GDPH	ZJH	FAHKMU	PHJM
N	1300	104	175	115	100
DME, N (%)	183 (14.08)	11 (10.58)	49 (28.00)	19 (16.52)	20 (20.00)
Age, year	55.50 ± 12.36	59.52 ± 13.07	56.04 ± 11.63	53.51 ± 12.63	58.99 ± 12.18
Gender, N (%)					
Men	782 (60.15)	66 (63.46)	107 (61.14)	75 (65.22)	55 (55.00)
Women	518 (39.85)	38 (36.54)	68 (38.86)	40 (34.78)	45 (45.00)
SBP, mmHg	131.41 ± 20.00	133.35 ± 20.87	137.54 ± 20.39	121.67 ± 17.92	136.83 ± 20.21
DBP, mmHg	76.99 ± 11.74	82.47 ± 11.12	79.30 ± 11.36	78.48 ± 11.84	80.87 ± 12.45
Hypertension, N (%)					
No	737 ± 56.69	57 (54.81)	92 (52.57)	69 (60.00)	50 (50.00)
Yes	563 ± 43.31	47 (45.19)	83 (47.43)	46 (40.00)	50 (50.00)
HbA1c, %	9.80 ± 2.89	9.27 ± 2.25	8.99 ± 2.56	9.02 ± 2.26	9.98 ± 5.21
Duration of DM, year	7.85 ± 7.25	9.79 ± 8.36	6.99 ± 6.19	8.33 ± 6.86	7.31 ± 7.07
HCT, %	39.62 ± 5.42	39.71 ± 4.99	39.66 ± 5.64	42.84 ± 4.64	41.57 ± 4.78
LDLc, mmol/L	3.27 ± 0.95	3.19 ± 0.94	3.11 ± 1.07	2.66 ± 0.92	2.96 ± 1.12
UACR stage, N (%)					
Stage 1	960 (60.69)	71 (68.27)	92 (52.57)	74 (64.35)	68 (68.00)
Stage 2	238 (18.31)	16 (15.38)	38 (21.71)	26 (22.61)	21 (21.00)
Stage 3	156 (12.00)	17 (16.35)	45 (25.71)	15 (13.04)	11 (11.00)

Data are mean ± standard deviation, or N (%). Abbreviations: DME, diabetic macular edema; DM, diabetes mellitus; SBP, systolic blood pressure; DBP, diastolic blood pressure; LDLc, low-density lipoprotein cholesterol; HCT, hematocrit; HbAlc, glycosylated hemoglobin; UACR, urine albumin-to-creatinine ratio; GDPH, the Guangdong Provincial People’s Hospital; ZJH, the Zhujiang Hospital of Southern Medical University; PHJM, People’s Hospital of JiangMen; FAHKMU, the First Affiliated Hospital of Kunming Medical University

In the training set with 1300 T2DM patients, a total of 183 (14.08%) cases of DME cases were documented. Baseline characteristics and distributions of the DME group and non-DME group were shown in Supplemental Table S3 and Figure S1. There was no significant difference between the two groups in age, sex, cardiovascular disease, TG and PLT (all *P* > 0.05). The DME group had higher values in SBP, DBP, TC, LDLc, HDLc, WBC, BUN, UA, SCr, and duration of DM (all *P* < 0.05), and lower values in RBC, Hb, HCT, and eGFR (all *P* < 0.05). Moreover, the DME group was more likely to have hypertension, higher UACR stage, HbA1c ≥ 8%, as well as DM and hypertension medication (all *P* < 0.05).

### Systemic variables selection

In the univariate logistic regression, 19 variables were significantly associated DME in the training set (Table 2). There is no collinearity between these variables in the multicollinearity test. These variables were included in the FSMLR analysis, and 6 factors, including the duration of DM, DBP, HCT, HDLc, HbA1c and UACR stage, were chosen to be candidate variables for DME prediction. However, HDLc was excluded from the final prediction model because of the result of HDLc was controversial to the literature and clinical common belief. In the end, the remaining five variables were included in the final model, and those variables were also approved by professional endocrinologists and ophthalmologists. Those five prediction factors for DME were presented in Table 3.

**Table 2:** Univariate logistic regression for DME risk in the training set

Variables	Odds ratio (95% CI)	*P* value
Age	1.00 (0.99–1.02)	0.454
Gender		
Men	[Reference]	
Women	1.03 (0.75–1.42)	0.860
BMI group		
<24	[Reference]	
24–28	0.80 (0.57–1.12)	**0.200**
≥28	0.60 (0.37–0.97)	**0.035**
SBP	1.03 (1.02–1.03)	**< 0.001**
DBP	1.02 (1.01–1.04)	**0.001**
Hypertension		
No	[Reference]	
Yes	1.89 (1.38–2.59)	**0.001**
Cardiovascular disease		
No	[Reference]	
Yes	1.02 (0.64–1.62)	0.934
TG	0.97 (0.90–1.06)	0.537
TC	1.16 (1.05–1.28)	**0.005**
LDLc	1.24 (1.06–1.46)	**0.009**
HDLc	1.76 (1.10–2.81)	**0.018**
RBC	0.40 (0.31–0.51)	**< 0.001**
Hb	0.97 (0.96–0.98)	**< 0.001**
HCT	0.87 (0.84–0.89)	**< 0.001**
WBC	1.06 (1.00-1.15)	**0.047**
PLT	1.00 (1.00-1.002)	0.782
BUN	1.01 (1.00-1.01)	**0.016**
UA	1.002 (1.00-1.003)	**0.008**
SCr	1.01 (1.00-1.01)	**< 0.001**
eGFR	0.98 (0.97–0.98)	**< 0.001**
Proteinuria		
(-)/(±)	[Reference]	
(+~)	8.45 (5.90-12.09)	**< 0.001**
UACR stage		
Stage 1	[Reference]	
Stage 2	3.14 (2.07–4.77)	**< 0.001**
Stage 3	13.85 (9.22–20.82)	**< 0.001**
HbA1c	1.03 (0.98–1.08)	**0.203**
HbA1c ≥ 8%		
No	[Reference]	
Yes	1.74 (1.18–2.57)	**0.005**
Duration of DM	1.07 (1.05–1.09)	**< 0.001**
Duration of DM ≥ 10 years		
No	[Reference]	
Yes	2.62 (1.90–3.61)	**< 0.001**
Treatment for DM		
Non	[Reference]	
Non-insulin hypoglycemic drugs	2.85 (1.75–4.63)	**< 0.001**
Insulin	4.48 (2.34–8.52)	**< 0.001**
Both	3.65 (2.20–6.05)	**< 0.001**
Blood pressure medication		
No	[Reference]	
Yes	1.71 (1.25–2.34)	**0.001**

*Abbreviations* DME, diabetic macular edema; DM, diabetes mellitus; BMI, body mass index; SBP, systolic blood pressure; DBP, diastolic blood pressure; TG, triglycerides; TC, total cholesterol; LDLc, low-density lipoprotein cholesterol; HDLc, high-density lipoprotein cholesterol; RBC, red blood cells; Hb, hemoglobin; HCT, hematocrit; WBC, white blood cells; PLT, platelet count; BUN, blood urea nitrogen; UA, uric acid; SCr, serum creatinine; eGFR, estimated glomerular filtration rate; UACR, urine albumin-to-creatinine ratio; HbAlC, glycosylated hemoglobin. Boldface indicates statistical significance

**Table 3:** Forward stepwise multivariate logistic regression for DME risk in the training set

Variables	Coefficient (95% CI)	Odds ratio (95% CI)	*P* value
UACR stage			
Stage 1	[Reference]	[Reference]	
Stage 2	0.82 (0.38–1.26)	2.26 (1.46–3.51)	**< 0.001**
Stage 3	1.86 (1.38–2.33)	6.40 (3.98–10.28)	**< 0.001**
HbA1c	0.08 (0.02–0.14)	1.08 (1.02–1.14)	**0.005**
DBP	0.03 (0.01–0.04)	1.03 (1.01–1.05)	**< 0.001**
HCT	-0.09 (-0.12 to -0.05)	0.92 (0.88–0.95)	**< 0.001**
Duration of DM	0.05 (0.02–0.07)	1.05 (1.02–1.08)	**< 0.001**

*Abbreviations* DME, diabetic macular edema; DM, diabetes mellitus; UACR, urine albumin-to-creatinine ratio; DBP, diastolic blood pressure; HCT, hematocrit; HbAlC, glycosylated hemoglobin. Boldface indicates statistical significance

### Performance evaluation

**Fig. 2:**
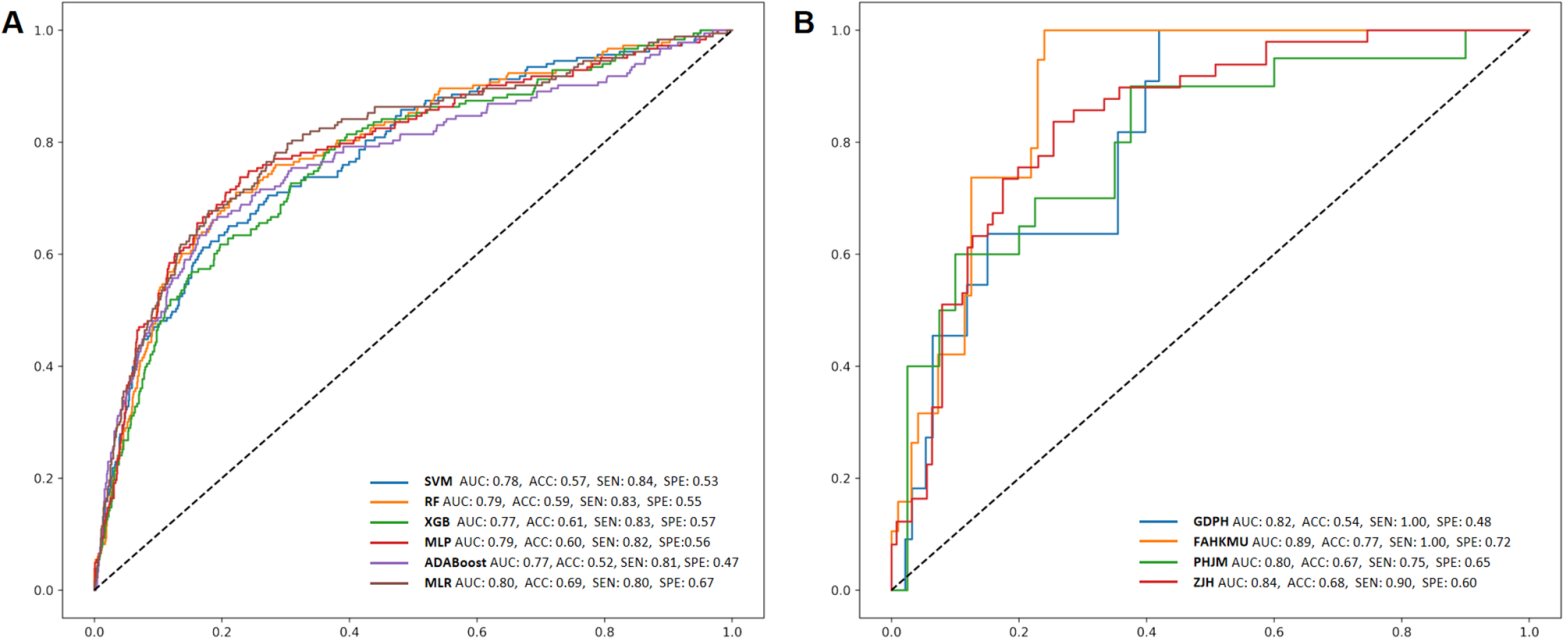
The Performance of the prediction models for identifying DME in T2DM patients. (**A**). Internal validation (five-fold cross validation): Machine Learning models with 26 system variables and MLR model with 5 system variables. These five systematic variables were selected by stepwise logistic regression. (**B**). We selected the MLR model with the best predictive performance for prospective validation from GDPH and external validation from 3 centers. Abbreviations: DME, diabetic macular edema; T2DM, Type 2 diabetes mellitus; AUC: area under receiver operating characteristic curve; ACC, accuracy; SEN, sensitivity; SPE, specificity; SVM, support Vector Mac; RF, random forest; XGB, extreme gradient boosting; MLP, multilayer perceptron; ADABOOST, adaptive boosting; MLR, multivariate logistic regression; GDPH, the Guangdong Provincial People’s Hospital; ZJH, the Zhujiang Hospital of Southern Medical University; PHJM, People’s Hospital of JiangMen; FAHKMU, the First Affiliated Hospital of Kunming Medical University

The MLR model using five risk factors was selected as the final prediction model due to its better performance than the machine learning model using all variables or the five variables (Fig. 2 and Supplemental Figure S2). The AUC, ACC, sensitivity, and specificity were 0.80, 0.69, 0.80, and 0.67 in the internal validation, and 0.82, 0.54, 1.00, and 0.48 in prospective validation, respectively. In external validation, the AUC, ACC, sensitivity and specificity were 0.84, 0.68, 0.90 and 0.60 in ZJH, 0.89, 0.77, 1.00 and 0.72 in FAHKMU, and 0.80, 0.67, 0.75, and 0.65 in PHJM, respectively.

We also developed an DME risk score based on the β-Coefficients of the five variables (Supplemental Table S4). The total score for the DME risk score was 10 points. The estimated DME risk for each score was shown in Supplemental Table S5. For the DME risk score, the AUC, ACC, sensitivity, and specificity were 0.80, 0.57, 0.87, and 0.52 in the internal validation, and 0.79, 0.43, 1.00, and 0.37 in prospective validation, respectively. In external validation, the AUC, ACC, sensitivity and specificity were 0.83, 0.65, 0.94 and 0.63 in ZJH, 0.85, 0.64, 1.00 and 0.58 in FAHKMU, and 0.76, 0.57, 0.80, and 0.51 in PHJM, respectively. In the association analysis, the odds ratios (ORs) (95% CI) for DME associated with the DME risk score were 1.68 (1.56–1.81) in the training set, 1.63 (1.21–2.19) in prospective validation set, and 1.67 (1.41–1.97) in ZJH dataset, 1.83 (1.40–2.41) in FAHKMU dataset, and 1.64 (1.28–2.11) in PHJM dataset, respectively. Moreover, T2DM patients with high DME risk (≥ 5 points) were 4.28-12.00 times more likely to have DME than those with low DME risk (< 5 points). More details about the performance evaluation of the DME risk score were presented in Supplemental Table S6 and Figure S3.

## Discussion

Early screening for DME is the key for preventing severe visual impairment in DM patients. In this multi-center study, we developed a prediction model for early identification of DME in patients with T2DM using 5 easily accessible systemic variables, namely duration of DM, DBP, HCT, HbA1c and UACR stage. This DME prediction model was validated in different datasets, and it maintained a stable and satisfactory performance with an AUC of over 0.80, highlighting the generalizability and reliability of the prediction model. With this model, efficiency of DME diagnosis in general practice or community-based clinic can be greatly improved. The model may also be applied to areas short of ophthalmic specialists or devices for early detection and treatment of DME.

Most previous studies on DME focused on analyzing the risk factors [27]–[29], while only a few of them evaluated the predictive power of these risk factors or developed a prediction model. For example, a cross-sectional study of 142 eyes from Spanish diabetic patients found that HbA1c, foveal thickness, and visual acuity were associated with DME, and a scoring system was constructed accordingly [19]. Besides, plasma cytokines, including platelet-derived growth factor-BB, tissue inhibitors of metalloproteinase-1, angiopoietin, and VEGFR-2, were found to be associated with DME in a study with a pilot cohort of 18 patients and a validation cohort of 200 patients. Based on the 4 plasma cytokines, the study also developed a dynamic nomogram to predict DME in T2DM patients [18]. Recently, in a cross-sectional study of 349 DM patients, 6 clinical features, including the presence of diabetic peripheral neuropathy (DPN) symptoms, uric acid, use of insulin only or not for treatment, insulin dosage, urinary protein grade, and disease duration, were chosen for a DME prediction nomogram [20]. All these models were deemed to be sufficiently accurate as our model. However, these studies only included limited samples and did not perform external validation, which may lead to biased results. Moreover, some clinical variables included in these models required special measurement methods, such as foveal thickness, plasma cytokines, and DPN symptom assessments, which would limit their clinical application in routine clinical visits. Our study overcame these shortcomings by a large sample size, multiple-center validation, and inclusion of systemic variables (duration of DM, DBP, HCT, HbA1c, and UACR stage) which can be easily accessed in community hospitals in the current DME prediction model.

Our results demonstrated that UACR stage was associated with DME, and a higher UACR stage indicated an increased risk of DME. In fact, UACR stage is the most important factor with the highest weight in the DME prediction model. This result is consistent with previous epidemiological studies that have examined the association between renal injury and DME, which showed that microalbuminuria and macroalbuminuria were risk factors for DME, with macroalbuminuria carrying a stronger risk [25], [30], [31]. Meanwhile, the pathological mechanism of DME is related to vascular hyperpermeability and leakage, which provides insight into our findings. A low serum protein concentration caused by proteinuria may reduce the plasma colloidal osmotic pressure, increasing edema and fluid retention according to Starling’s Eq. [32] Thus, patients with macroalbuminuria may be prone to have more fluid leakage from the damaged retinal vessels, which leads to DME eventually.

Another important risk factor for DME in the MLR model was HCT, but the relationship between DME and HCT has been explored only in a handful of previous studies. A cross-sectional, case-control study of 312 T2DM patients showed that there were lower HCT levels in individuals with severe DR or DME [26]. It was also observed that ellipsoid zone disruption in DME patients was associated with decreased HCT [33]. Similarly, HCT might correlated with increased macular retinal thickness in diabetic patients. Furthermore, HCT is the ratio of the volume of red blood cells in the blood and related to anemia, and anemia was showed to be associated with an increased risk of DME in an observational study of 306 patients with T2DM [34]. Anemia is accompanied by a decrease in blood oxygen carrying capacity, resulting in insufficient oxygen supply to the retina. Long-term hypoxia will cause microvascular endothelial damage and increase permeability, which are crucial in the pathogenesis of DME [35]. Overall, HCT may be a useful indicator for monitoring DME status. The relationship between HCT and DME should be well elucidated in large-scale prospective clinical trials and animal experiments in the future.

In addition, glycemic control status, hypertension, and the duration of diabetes are recognized as the essential risk factors for DME. Indicators related to these factors were also selected by the model as crucial risk factors for DME in our study. As a marker of glycemic control status over the past three months, HbA1c was significantly associated with retinopathy progression, including DME, in DM patients. [1], [16], [27], [36], [37] It has been demonstrated that the risk of DME was higher in DM patients with HbA1c levels > 8.0% [29], [38]. Furthermore, clinical trials indicated that early intensive glucose control may be beneficial in preventing DR and DME in both type 1 and type 2 DM patients [36], [39], [40]. Besides, hypertension is another modifiable risk factor for DME. Hypertension is believed to compromise vascular permeability in the already-damaged diabetic vasculature by increasing the perfusion pressure in retinal vessels, leading to DME and retinal hemorrhage. Our study showed a significant association between DBP and DME. Moreover, some previous studies have reported that both DBP and SBP [37], or only SBP [41], [42] were significantly associated with DME. The above studies all highlighted the undeniable risks posed by elevated blood pressure, and blood pressure-lowering treatment may reduce the occurrence of macular edema and arteriovenous nicking [43]. Therefore, it is important to monitor and manage hypertension in DM patients in order to prevent exacerbations of retinal damage and DME.

This study constructed and validated a prediction model that enables physicians to distinguish patients at high risk for DME who should be referred to an ophthalmologist in the absence of ophthalmic evaluations such as visual acuity and fundus examinations. To apply the model in daily scenarios and increase the understanding of risk factors in the model, we transformed the prediction model into a simple risk score. The score maintained good predictive performance. Based on the prediction model or risk score, endocrinologists, community doctors, primary care doctors, and other non-ophthalmic physicians could easily conduct DME risk stratification and screening for T2DM patients. A patient predicted to be at high risk should be referred to the ophthalmic specialist for further examinations. On the other hand, if a patient is found to be at a low risk of DME, physicians should take appropriate measures to control the modifiable risk factors for DME, such as lowering blood pressure, controlling blood glucose levels, improving anemia, or reducing urinary albumin, to prevent the occurrence of DME. The DME risk score can be used by physicians, and may even become a self-assessment and risk management tool for T2DM patients in the future. Meanwhile, we are preparing to incorporate this model into the CDSS system to automatically capture the information of the 5 system variables and notify the physicians whether the patient is at high risk of DME. This DME detection model based on systemic variables may also have some potential clinical benefits. Firstly, DME usually appears in the eyes of patients with DR [44], which means that by detecting DME, we may also identify high-risk patients with DR. In addition, some systemic parameters such as HbA1c, DBP, and HCT are associated not only with DME, but also with other eye diseases such as retinal vein occlusion [45], [46]. Therefore, it is possible to find other eye diseases by evaluating these risk factors. In conclusion, the model’s ability to detect DME not only helps in the early identification and management of DME itself, but also provides an opportunity to identify DR and other eye diseases simultaneously.

The strengths of the present study include a large sample size and a multi-center population. In addition, the 5 systematic variables included in the model are all easily available clinical data, which makes the utility of the prediction model and risk score practical in clinical practice. There are also some limitations in this study. Firstly, the DME prediction model was developed only to identify patients with DME, and prediction model on the severity of DME needs to be further explored. Secondly, we only included patients with T2DM, and the prediction of DME in patients with type 1 DM needs to be further investigated. Thirdly, due to practical constraints, not all patients underwent Optical Coherence Tomography (OCT) examination. Therefore, we diagnosed DME according to the ETDRS report, which may have affected the comprehensive evaluation of certain aspects related to DME diagnosis. However, we validated the accuracy of DME diagnosis in patients who underwent OCT examination. Fourthly, the clinical utility of this model lies in its ability to facilitate the detection of DME specifically by non-ophthalmic healthcare professionals, without factoring in DR grade or visual acuity. This may come at a sacrifice to the accuracy of the model. Finally, this is a hospital-based study, and further studies are needed to determine if the prediction model is applicable as a screening tool in general DM populations.

In conclusion, we have developed a simple and reliable prediction model for rapid identification of DME in patients with T2DM using 5 easily available systemic variables. The model is promising to be routinely used by non-ophthalmic physicians in community-based practice or in remote areas to quickly identify DM patients with high risk of DME, thus promoting early diagnosis and treatment of DME.

### Electronic supplementary material

Below is the link to the electronic supplementary material.

Supplementary Material 1

## Data Availability

The data used during the current study are available from the corresponding authors on reasonable request.
