# Supplementary material for: Development and validation of a simple and practical model for early detection of diabetic macular edema in patients with type 2 diabetes mellitus using easily accessible systemic variables
Source: J Transl Med. 2024 May 31;22:523. doi: 10.1186/s12967-024-05328-y (PMC11140894; doi:10.1186/s12967-024-05328-y)
Supplement: Supplementary file 1 — Supplementary Material 1 [file 12967_2024_5328_MOESM1_ESM.docx]

**Supplementary Online Content**

Figure S1. Variable distribution for T2DM patients with and without DME in the training set.

Figure S2. Performance of the machine learning models and logistic regression model using five systemic variables for identifying DME in T2DM patients.

Figure S3. The receiver operating characteristic (ROC) curves of the DME risk score for identifying DME in T2DM patients.

Table S1. Association of visual acuity and DR grade with DME in T2DM patients.

Table S2. Association between systemic variables and DR in T2DM patients.

Table S3. Baseline characteristics stratified by DME status in the training set.

Table S4. Risk scoring for DME risk in T2DM patients in the training set.

Table S5. The estimated risk of DME corresponding to the total score.

Table S6. Association between DME risk score and DME in T2DM patients.

**
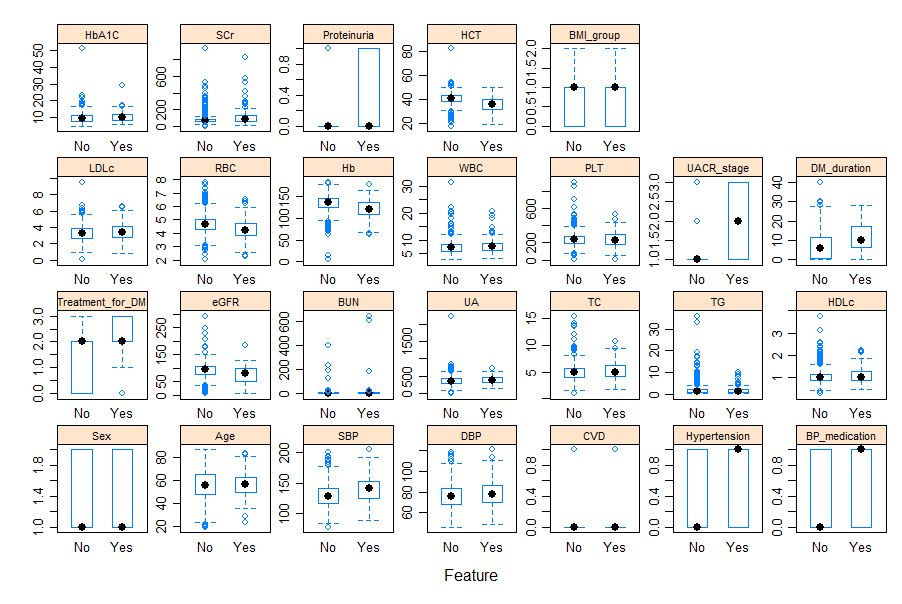
**

**Figure S1. Variable distribution for T2DM patients with and without DME in the training set.** Abbreviations: DME, diabetic macular edema; T2DM, Type 2 [diabetes mellitus](javascript:;); BMI, body mass index; SBP, systolic blood pressure; DBP, diastolic blood pressure; TG, triglycerides; TC, total cholesterol; LDLc, low-density lipoprotein cholesterol; HDLc, high-density lipoprotein cholesterol; RBC, red blood cells; Hb, hemoglobin; HCT, hematocrit; WBC, white blood cells; PLT, platelet count; BUN, blood urea nitrogen; UA, [uric acid](javascript:;); SCr, serum creatinine; eGFR, estimated glomerular filtration rate; UACR, urine albumin-to-creatinine ratio; HbAlC, glycosylated hemoglobin; CVD, cardiovascular disease.


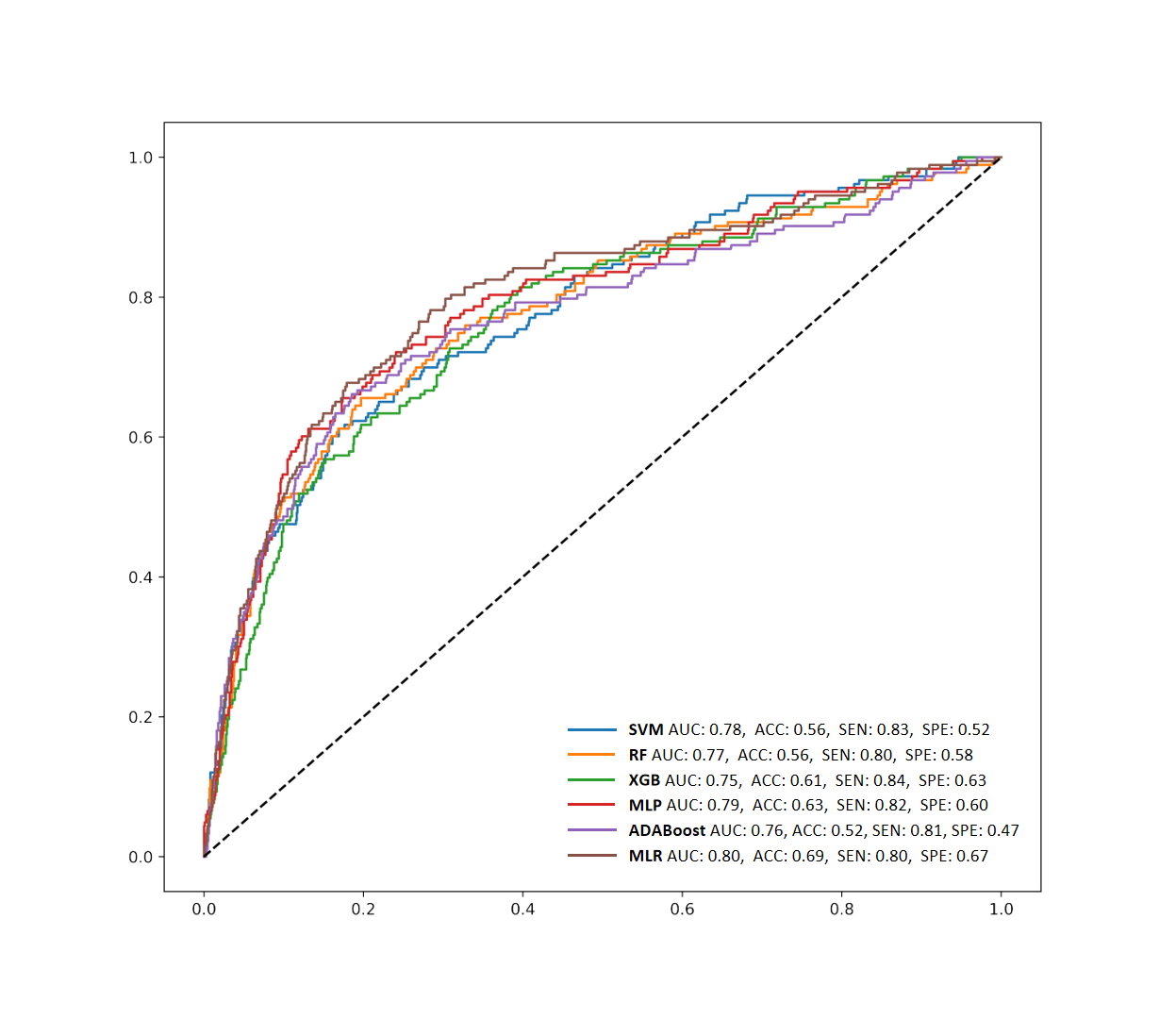


**Figure S2.** **Performance of the** **machine learning models and logistic regression model using five systemic variables for identifying DME in T2DM patients.** Abbreviations: DME, diabetic macular edema; T2DM, Type 2 [diabetes mellitus](javascript:;); AUC: area under receiver operating characteristic curve; ACC, accuracy; SEN, sensitivity; SPE, specificity; SVM, support Vector Mac; RF, random forest; XGB, extreme gradient boosting; MLP, multilayer perceptron; ADABOOST, adaptive boosting; MLR, multivariate logistic regression; GDPH, the Guangdong Provincial People’s Hospital; ZJH, the Zhujiang Hospital of Southern Medical University; PHJM, People's Hospital of JiangMen; FAHKMU, the First Affiliated Hospital of Kunming Medical University.


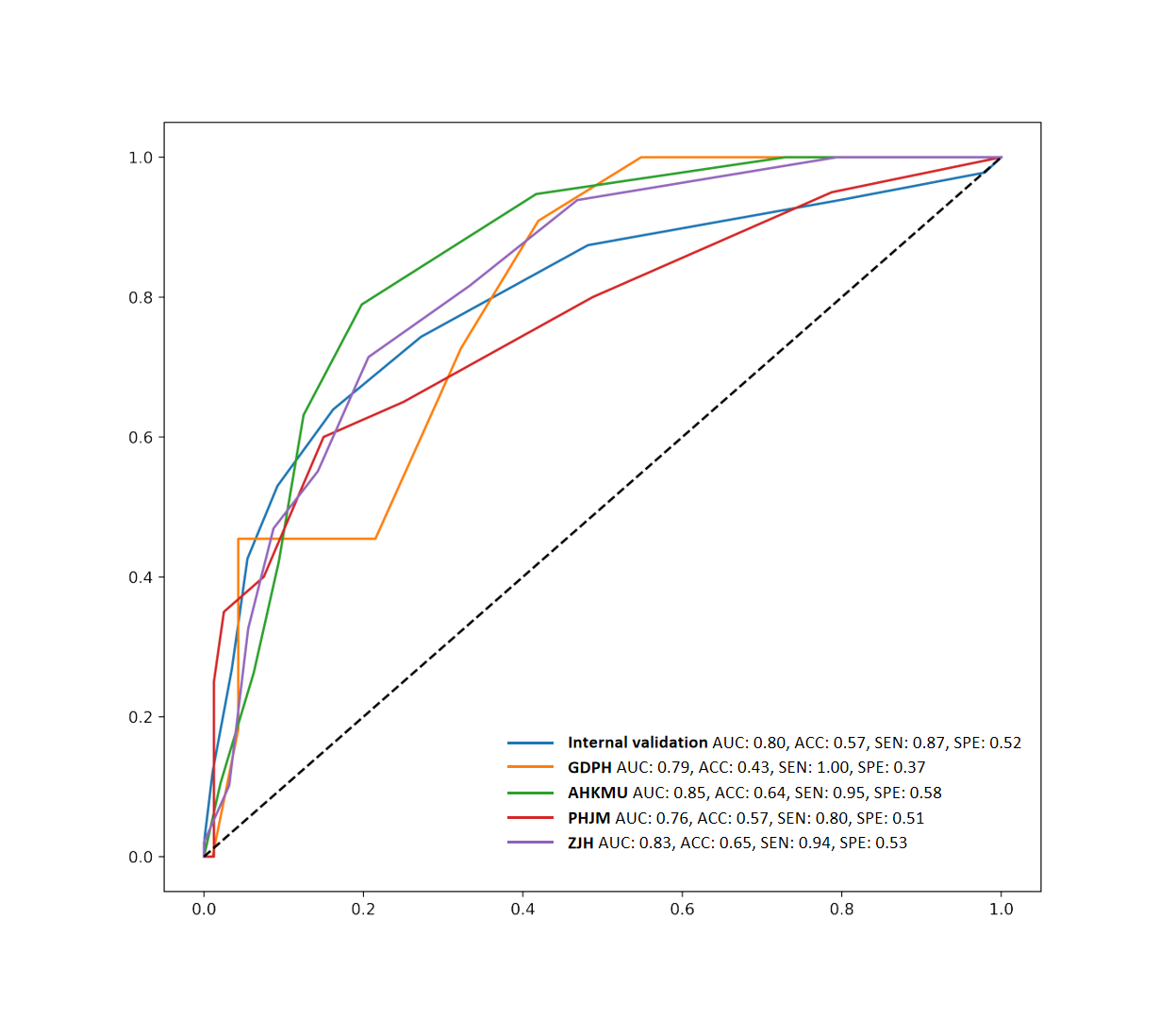


**Figure S3. The receiver operating characteristic (ROC) curves of the** **DME risk score for identifying DME in T2DM patients.** Abbreviations: DME, diabetic macular edema; T2DM, Type 2 [diabetes mellitus](javascript:;); AUC: area under receiver operating characteristic curve; ACC, accuracy; SEN, sensitivity; SPE, specificity; GDPH, the Guangdong Provincial People’s Hospital; ZJH, the Zhujiang Hospital of Southern Medical University; PHJM, People's Hospital of JiangMen; FAHKMU, the First Affiliated Hospital of Kunming Medical University.

**Table S1. Association of visual acuity and DR grade with DME in T2DM patients.**

|  | Odds ratio (95% CI) | P value |
| --- | --- | --- |
| Visual acuity (Best corrected) | 0.16 (0.00–0.23) | **<0.001** |
| DR stage |  |  |
| NPDR | [Reference] |  |
| PDR | 2.78 (1.56-3.37) | **0.008** |

Abbreviations: DME, diabetic macular edema; DM, diabetes mellitus; DR, diabetic retinopathy; NPDR, Non-proliferative diabetic retinopathy; PDR, Proliferative diabetic retinopathy; BMI, body mass index; SBP, systolic blood pressure; DBP, diastolic blood pressure; TG, triglycerides; TC, total cholesterol; LDLc, low-density lipoprotein cholesterol; HDLc, high-density lipoprotein cholesterol; RBC, red blood cells; Hb, hemoglobin; HCT, hematocrit; WBC, white blood cells; PLT, platelet count; BUN, blood urea nitrogen; UA, uric acid; eGFR, estimated glomerular filtration rate; UACR, urine albumin-to-creatinine ratio; HbAlC, glycosylated hemoglobin. Logistic regression model adjusted for age, gender, BMI, SPB, DPB, hypertension, cardiovascular disease, TG, TC, LDLc, HDLc, RBC, Hb, HCT, WBC, PLT, BUN, UA, eGFR, UACR stage, HbA1c, duration of DM, treatment for DM, blood pressure medication. Boldface indicates statistical significance.

**Table S2. Association between systemic variables and DR in T2DM patients.**

| Variables | Odds ratio (95% CI) | P value |
| --- | --- | --- |
| Age | 0.67 (0.43-1.03) | 0.070 |
| Gender |  |  |
| Men | [Reference] |  |
| Women | 0.97 (0.95-0.99) | **0.007** |
| BMI | 0.95 (0.89-1.00) | 0.052 |
| SBP | 1.01 (0.99-1.02) | 0.217 |
| DBP | 1.02 (0.99-1.04) | 0.170 |
| Hypertension |  |  |
| No | [Reference] |  |
| Yes | 1.82 (0.78-4.25) | 0.164 |
| Cardiovascular disease |  |  |
| No | [Reference] |  |
| Yes | 1.07 (0.60-1.89) | 0.829 |
| TG | 0.85 (0.71-1.01) | 0.059 |
| TC | 1.40 (0.85-2.29) | 0.185 |
| LDLc | 0.86 (0.47-1.59) | 0.638 |
| HDLc | 1.12 (0.47-2.68) | 0.795 |
| RBC | 1.30 (0.84-2.02) | 0.246 |
| Hb | 1.02 (0.98-1.05) | 0.331 |
| HCT | 0.84 (0.74-0.96) | **0.010** |
| WBC | 1.10 (1.01-1.19) | **0.021** |
| PLT | 0.99 (0.99-0.99) | **0.004** |
| BUN | 1.00 (0.99-1.01) | 0.272 |
| UA | 1.00 (0.99-1.002) | 0.569 |
| eGFR | 0.99 (0.99-1.01) | 0.693 |
| UACR stage |  |  |
| Stage 1 | [Reference] |  |
| Stage 2 | 2.02 (1.26-3.23) | **0.003** |
| Stage 3 | 4.78 (2.67-8.54) | **<0.001** |
| HbA1c | 1.10 (1.03-1.17) | **0.002** |
| Duration of DM | 1.06 (1.03-1.10) | **<0.001** |
| Treatment for DM |  |  |
| Non | [Reference] |  |
| Non-insulin hypoglycemic drugs | 1.31 (0.57-3.04) | 0.529 |
| Insulin | 2.23 (1.24-4.00) | **0.007** |
| Both | 1.99 (1.04-3.81) | **0.038** |
| Blood pressure medication |  |  |
| No | [Reference] |  |
| Yes | 1.06 (1.03-1.10) | 0.353 |

Abbreviations: DME, diabetic macular edema; DM, diabetes mellitus; DR, diabetic retinopathy; BMI, body mass index; SBP, systolic blood pressure; DBP, diastolic blood pressure; TG, triglycerides; TC, total cholesterol; LDLc, low-density lipoprotein cholesterol; HDLc, high-density lipoprotein cholesterol; RBC, red blood cells; Hb, hemoglobin; HCT, hematocrit; WBC, white blood cells; PLT, platelet count; BUN, blood urea nitrogen; UA, uric acid; eGFR, estimated glomerular filtration rate; UACR, urine albumin-to-creatinine ratio; HbAlC, glycosylated hemoglobin. Multivariate logistic regression was used to explore the association between systemic variables and DR. Boldface indicates statistical significance.

**Table S3. Baseline characteristics stratified by DME status in the training set.**

| Baseline characteristics | Total | DME | Non-DME | P value |
| --- | --- | --- | --- | --- |
| N | 1041 | 147 (14.12) | 894 (85.88) |  |
| Age, year | 55.50±12.36 | 56.13±10.72 | 55.40±12.60 | 0.455 |
| Gender, N (%) |  |  |  | 0.860 |
| Men | 782 (60.15) | 109 (59.56) | 673 (60.25) |  |
| Women | 518 (39.85) | 74 (40.44) | 444 (39.75) |  |
| BMI group, N (%) |  |  |  | 0.087 |
| <24 | 541 (41.62) | 88 (48.09) | 453 (40.56) |  |
| 24-28 | 520 (40.00) | 70 (38.25) | 450 (40.29) |  |
| ≥28 | 239 (18.38) | 25 (13.66) | 214 (19.16) |  |
| SBP, mmHg | 131.41±20.00 | 140.77±22.90 | 129.89±19.25 | **<0.001** |
| DBP, mmHg | 76.99±11.74 | 79.77±12.43 | 76.54±11.56 | **0.001** |
| Hypertension, N (%) |  |  |  | **<0.001** |
| No | 737 (56.69) | 79 (43.17) | 658 (58.91) |  |
| Yes | 563 (43.31) | 104 (56.83) | 459 (41.09) |  |
| Cardiovascular disease, N (%) |  |  |  | 0.934 |
| No |  |  |  |  |
| Yes |  |  |  |  |
| TG, mmol/L | 2.07±2.18 | 1.98±1.65 | 2.09±2.26 | 0.537 |
| TC, mmol/L | 5.01±1.55 | 5.29±1.55 | 4.97±1.38 | **0.004** |
| LDLc, mmol/L | 3.27±0.95 | 3.44±1.06 | 3.24±0.93 | **0.008** |
| HDLc, mmol/L | 1.05±0.31 | 1.13±0.34 | 1.04±0.31 | **0.017** |
| RBC, /L | 4.62±0.70 | 4.26±0.74 | 4.68±0.68 | **<0.001** |
| Hb, g/L | 133.22±19.56 | 121.37±21.46 | 135.16±18.53 | **<0.001** |
| HCT,% | 39.62±5.42 | 36.01±6.15 | 40.21±5.05 | **<0.001** |
| WBC, /L | 7.41±2.32 | 7.73±2.54 | 7.36±2.28 | **0.045** |
| PLT, /L | 243.86±77.16 | 242.40±80.48 | 244.10±76.64 | 0.783 |
| BUN, mmol/L | 8.05±28.78 | 16.02±66.10 | 6.75±15.47 | **<0.001** |
| UA, umol/L | 376.03±123.71 | 400.26±111.71 | 372.06±125.17 | **0.004** |
| SCr, umol/L | 83.30±58.54 | 112.54±93.56 | 78.51±48.97 | **<0.001** |
| eGFR, ml/min/1.73m² | 89.70±28.41 | 74.80±32.15 | 92.14±26.99 | **<0.001** |
| [Proteinuria](javascript:;), N (%) |  |  |  | **<0.001** |
| (-)/(±) | 1123 (86.38) | 102 (55.74) | 1021 (91.41) |  |
| (+~) | 177 (13.62) | 81 (44.26) | 96 (8.59) |  |
| UACR stage, N (%) |  |  |  | **<0.001** |
| Stage 1 | 906 (69.69) | 61 (33.33) | 845 (75.65) |  |
| Stage 2 | 238 (18.31) | 44 (24.04) | 194 (17.37) |  |
| Stage 3 | 156 (12.00) | 78 (42.62) | 78 (6.98) |  |
| HbA1c, % | 9.80±2.89 | 10.06±2.67 | 9.76±2.93 | 0.200 |
| HbA1c ≥ 8%, N (%) |  |  |  | **0.005** |
| No | 361 (27.77) | 35 (19.13) | 326 (29.19) |  |
| Yes | 939 (72.23) | 148 (80.87) | 791 (70.81) |  |
| [Duration of](javascript:;) DM, year | 7.85±7.25 | 11.20±7.07 | 7.30±7.14 | **<0.001** |
| [Duration of](javascript:;) DM ≥ 10 years, N (%) |  |  |  | **<0.001** |
| No | 789 (60.69) | 74 (40.44) | 715 (64.01) |  |
| Yes | 511 (59.56) | 109 (59.56) | 402 (35.99) |  |
| Treatment for DM, N (%) |  |  |  | **<0.001** |
| Non | 381 (29.31) | 23 (12.57) | 358 (32.05) |  |
| Non-insulin hypoglycemic drugs | 504 (38.77) | 78 (42.62) | 426 (38.14) |  |
| [Insulin](javascript:;) | 94 (7.23) | 21 (11.48) | 73 (6.54) |  |
| Both | 321 (24.69) | 61 (33.33) | 260 (23.28) |  |
| Blood pressure medication, N (%) |  |  |  | **0.001** |
| No | 793 (61.00) | 91 (49.73) | 702 (62.85) |  |
| Yes | 507 (39.00) | 92 (50.27) | 415 (37.15) |  |

Data are mean±standard deviation, or N (%). Abbreviations: DME, diabetic macular edema; BMI, body mass index; SBP, systolic blood pressure; DBP, diastolic blood pressure; TG, triglycerides; TC, total cholesterol; LDLc, low-density lipoprotein cholesterol; HDLc, high-density lipoprotein cholesterol; RBC, red blood cells; Hb, hemoglobin; HCT, hematocrit; WBC, white blood cells; PLT, platelet count; BUN, blood urea nitrogen; UA, [uric acid](javascript:;); SCr, serum creatinine; eGFR, estimated glomerular filtration rate; UACR, urine albumin-to-creatinine ratio; HbAlC, glycosylated hemoglobin; DM, [diabetes mellitus](javascript:;); CVD, cardiovascular disease; DME, diabetic macular edema.

All P values were calculated using the t-test for continuous variables and the χ^2^ test for categorical variables. Boldface indicates statistical significance.

**Table S4. Risk scoring for DME risk in T2DM patients in the training set.**

| Variables | Coefficient (95% CI) | Odds ratio (95% CI) | P value | **Score** |
| --- | --- | --- | --- | --- |
| UACR stage |  |  |  |  |
| Stage 1 | [Reference] | [Reference] |  | 0 |
| Stage 2 | 0.84 (0.41-1.28) | 2.32 (1.50-3.59) | <0.001 | 2 |
| Stage 3 | 1.92 (1.45-2.39) | 6.83 (4.26-10.93) | <0.001 | 4 |
| HbA1c |  |  |  |  |
| <8 | [Reference] | [Reference] |  | 0 |
| ≥8 | 0.67 (0.22-1.11) | 1.95 (1.25-3.04) | 0.003 | 1 |
| DBP |  |  |  |  |
| <70 | [Reference] | [Reference] |  | 0 |
| 70-90 | 0.44 (0.00-0.88) | 1.55 (1.00-2.41) | 0.051 | 1 |
| ≥90 | 0.92 (0.35-1.49) | 2.51 (1.42-4.46) | 0.002 | 2 |
| HCT |  |  |  |  |
| >38 | [Reference] | [Reference] |  | 0 |
| 35-38 | 0.61 (0.14-1.09) | 1.85 (1.15-2.97) | 0.012 | 1 |
| ≤35 | 1.17 (0.70-1.65) | 3.23 (2.01-5.19) | <0.001 | 2 |
| [Duration of](javascript:;) DM |  |  |  |  |
| <10 years | [Reference] | [Reference] |  | 0 |
| ≥10 years | 0.69 (0.33-1.05) | 1.99 (1.50-3.59) | <0.001 | 1 |
| **Total** |  |  |  | 10 |

Abbreviations: DME, diabetic macular edema; T2DM, Type 2 [diabetes mellitus](javascript:;); UACR, urine albumin-to-creatinine ratio; DBP, diastolic blood pressure; HCT, hematocrit; HbAlC, glycosylated hemoglobin. Boldface indicates statistical significance.

**Table S5. The estimated risk of DME corresponding to the total score.**

| Point total (DME risk score) | Estimate of risk |
| --- | --- |
| 0 | 0.0177 |
| 1 | 0.0288 |
| 2 | 0.0466 |
| 3 | 0.0746 |
| 4 | 0.1173 |
| 5 | 0.1797 |
| 6 | 0.2653 |
| 7 | 0.3732 |
| 8 | 0.4954 |
| 9 | 0.6181 |
| 10 | 0.7274 |

Abbreviations: DME, diabetic macular edema.

**Table S6. Association between DME risk score and DME in T2DM patients.**

|  | **Internal validation** | **Prospective validation** | **External validation** | | |
| --- | --- | --- | --- | --- | --- |
|  | **GDPH** | **GDPH** | **ZJH** | **FAHKMU** | **PHJM** |
|  | Odds ratio (95% CI) | Odds ratio (95% CI) | Odds ratio (95% CI) | Odds ratio (95% CI) | Odds ratio (95% CI) |
| **Total score** | 1.68 (1.56-1.81) | 1.63 (1.21-2.19) | 1.67 (1.41-1.97) | 1.83 (1.40-2.41) | 1.64 (1.28-2.11) |
| **Performance** |  |  |  |  |  |
| **AUC** | **0.80** | **0.79** | **0.83** | **0.85** | **0.76** |
| ACC | 0.57 | 0.43 | 0.65 | 0.64 | 0.57 |
| Specificity | 0.52 | 0.37 | 0.53 | 0.58 | 0.51 |
| Sensitivity | 0.87 | 1.00 | 0.94 | 0.95 | 0.80 |
| NPV | 0.96 | 0.49 | 0.96 | 0.98 | 0.91 |
| PPV | 0.23 | 0.32 | 0.44 | 0.31 | 0.29 |
| **Risk level** |  |  |  |  |  |
| Low risk (0-4) | [Reference] | [Reference] | [Reference] | [Reference] | [Reference] |
| High risk (5-10) | 9.17 (6.52-12.89) | 4.28 (1.16-15.82) | 9.62 (4.52-20.46) | 12.00 (3.95-36.46) | 8.50 (2.87-25.15) |
| **Performance** |  |  |  |  |  |
| **AUC** | **0.80** | **0.79** | **0.83** | **0.85** | **0.76** |
| ACC | 0.81 | 0.70 | 0.77 | 0.83 | 0.80 |
| Specificity | 0.84 | 0.71 | 0.79 | 0.88 | 0.85 |
| Sensitivity | 0.64 | 0.64 | 0.71 | 0.63 | 0.60 |
| NPV | 0.93 | 0.94 | 0.88 | 0.92 | 0.89 |
| PPV | 0.39 | 0.21 | 0.57 | 0.50 | 0.50 |

All the P <0.01. Abbreviations: DME, diabetic macular edema; T2DM, Type 2 [diabetes mellitus](javascript:;); PPV, positive predictive value; NPV, negative predictive value; GDPH, the Guangdong Provincial People’s Hospital; ZJH, the Zhujiang Hospital of Southern Medical University; PHJM, People's Hospital of JiangMen; FAHKMU, the First Affiliated Hospital of Kunming Medical University.
